# Caloropathy

**Published:** 1877-02

**Authors:** 


### Editorial.

### CALOROPATHY.

In all pursuits it is but natural that men attempt to shorten the routine of duties, and lighten the burden of labor incident to their particular avocation. Physicians are by no means exempt from this inclination. Hence, one-ideal systems of medicine commenced long ago—have been in existence, time immemorial. All such have for their object the abridgement of professional studies and practical duties, and depend upon public credulity for success. Stahl's expectant system and Hahnemann's infinitessimalism, virtually ignore medicine entirely, while the various other *pathies* fix upon one remedy or particular plan of treatment for all manner of disease. Not only do we find this deplorable tendency in offshoots from the regular profession, but also to some extent with our most zealous practitioners of rational medicine. Patients are too often subjected to routine treatment, without giving them the advantages derived from a knowledge of the true pathological condition, obtained by critical, and, sometimes, laborious examinations. Not only so, but means which require time and physical and mental effort for their application, are sometimes sadly neglected. General bloodletting, local depletion and counter irritation are often lost sight of in the treatment of inflammatory diseases demanding these resources. We have been led to these reflections by the article on Turkish Bath by Dr. J. S. Wilson, found in this number of the JOURNAL. Ignoring the water of the Hydropath and the vapor of the *Steamer*, Dr. Wilson has found in heated dry air the panacea for all our woes!

To many of our readers the processes and general character of the Turkish Bath are unknown, and while for the information of such, we give the Doctor's description of it, we of course recognise in it a species of the genus *pathy*, so far as its universal application in the cure of disease is concerned. We rate it with the pets of other pathies, and while for water, steam, heat and expectancy, we occasionally have a proper subject, we should greatly deplore the result of having patients indiscriminately subjected to any one alone. Secret remedies and patented nostrums run the same general schedule of the *pathies*. Large pay and little to do is the real motto of all.

